# Limited impacts of extensive human land use on dominance, specialization, and biotic homogenization in boreal plant communities

**DOI:** 10.1186/s12898-015-0037-9

**Published:** 2015-02-13

**Authors:** Stephen J Mayor, Stan Boutin, Fangliang He, James F Cahill

**Affiliations:** Department of Biological Science, University of Alberta, Edmonton, Alberta T6G 2E9 Canada; Department of Renewable Resources, University of Alberta, Edmonton, Alberta T6G 2H1 Canada

**Keywords:** Anthropogenic disturbance, Biodiversity, Biotic homogenization, Boreal forest, Community structure, Human land use, Niche width, Rank species occupancy curve, Specialization, Vascular plants

## Abstract

**Background:**

Niche theory predicts that human disturbance should influence the assembly of communities, favouring functionally homogeneous communities dominated by few but widespread generalists. The decline and loss of specialists leaves communities with species that are functionally more similar. Evenness of species occupancy declines, such that species become either widespread of rare. These patterns have often been observed, but it is unclear if they are a general result of human disturbance or specific to communities that are rich in species, in complex, spatially heterogeneous environments where the problem has often been investigated. We therefore tested whether human disturbance impacts dominance/evenness of species occupancy in communities, specialism/generalism of species, and functional biotic homogenization in the spatially relatively homogeneous, species poor boreal forest region of Alberta, Canada. We investigated 371 boreal vascular plant communities varying 0 – 100% in proportion of human land use.

**Results:**

Rank species occupancy curves revealed high species dominance regardless of disturbance: within any disturbance class a few species occupied nearly every site and most species were found in a low proportion of sites. However, species were more widespread and displayed more even occupancy in intermediately disturbed communities than among communities of either low or high disturbance. We defined specialists and generalists based on turnover in co-occupants and thereby assessed impacts of human disturbance on specialization of species and community homogenization. Generalists were not disproportionately found at higher disturbance sites, and did not occupy more sites. Communities with greater human disturbance were not more functionally homogeneous; they did not harbor communities with more generalists.

**Conclusions:**

We unexpectedly did not observe strong linkages between species specialism/generalism and disturbance, nor between community homogenization and disturbance. These results contrast previous findings in more species rich, complex or spatially heterogeneous systems and ecological models. We suggest that broad occupancy-based intercommunity patterns are insensitive to human land use extent in boreal vascular plants, perhaps because of ubiquity of generalists, low species richness, and history of natural disturbance. The poor sensitivity of these metrics to disturbance presents challenges for monitoring and managing impacts to biodiversity in this region.

## Background

Global loss of biodiversity is a major concern, and human land use is among its chief causes [1]. At local scales, species loss is less frequently observed, and the spread of weedy or exotic species often instead results in increases in richness [2]. Focusing on increased richness alone can however give the misleading impression that land use has negligible or positive effects on biodiversity, masking declines in species at larger scales and oversimplifying “biodiversity” to mere species counts, ignoring the composition of species and broader ecology of communities.

Communities may change in important ways with little impact on richness or diversity [3-5]. In some communities with expanded human land use, generalists may replace specialists, dominance by fewer species may increase, and communities may become more functionally homogeneous [6-9]. For instance, in metacommunity simulations comparing indices of biodiversity, specialists declined rapidly with disturbance intensity while species richness and diversity were far less sensitive to changes in disturbance [3].

We therefore explore two sets of complementary elements of community structure to test for impacts of anthropogenic disturbance. First, we explore distributions of occupancy (via ranked species occupancy curves), which indicate the relative occurrence of species across communities. Some species are present in many sites, while others occupy few sites. The distribution of species by occupancy is an important pattern in community ecology that, it has been suggested, has predictable shapes for disturbed and undisturbed communities [10]. Second, we investigate patterns of species on the specialist-generalist continuum, and biotic homogenization (the similarity of species across communities). A core ecological concept is that species vary not only in the conditions in which they exist, their niche, but in the range of conditions over which they exist, the breadth of their niche [11]. Theory suggests that disturbance poses greater threats, including extinction risk, to species with narrower niches (specialists) [12-14], and empirical evidence generally supports this expectation [3,7,15-17]. As a result, individual local communities tend to lose specialist species and retain or gain generalist species, leaving communities of more similar species composition following disturbance [7].

Motivation to better understand these aspects of biodiversity change with land use is three-fold. One, we seek to identify the ecological risks facing communities. Specialists, for example, play important roles in ecosystems and functional homogenization can reduce ecosystem function and resilience or resistance to further disturbance [7]. Low relative occupancy may contribute to extinction risk. Two, we seek to assess the potential utility of community metrics for ecological monitoring applications. If widespread, these patterns may serve as powerful local signatures of human land use on biodiversity not evident from species richness patterns, with utility as community-level metrics for ecological monitoring. And three, we test the generality of conceptual ideas of biodiversity change driven by disturbance. Disturbance, anthropogenic or natural, may have general predictable outcomes for ecological communities.

We investigate these patterns in a region unlike those in which these patterns are typically investigated: the boreal vascular plant communities of northern Alberta, Canada. In this large region, rapid expansion of human footprint, ongoing regional land use planning and heightened interest in broad scale environmental monitoring have deepened the importance of effective evaluation of how communities change with human land use at a regional scale. Also, spatial heterogeneity and species richness are both relatively low, and relative to previous studies of biotic homogenization, there are no major elevation or climate gradients.

### Occupancy

Among the most fundamental characteristics of communities is the relative abundance or occupancy among species. Akin to species abundance distributions, ranked species occupancy curves (RSOC)s provide detailed descriptions of the prevalence and rarity of species where abundance data are lacking [10]. RSOCs also retain species identity information and avoid arbitrary frequency binning that is characteristic of occupancy frequency distributions [10].

Jenkins [10] built on the intermediate disturbance hypothesis [18] and studies of species occupancy [19-22] to develop hypotheses for RSOC shapes, suggesting those shapes should vary along a successional gradient, and as a result, on a spatial gradient in disturbance. He suggested that with high disturbance, recruitment limitation should cause an exponential RSOC where some disturbance-adapted species are prevalent but most others rare. At intermediate disturbance, Jenkins predicted a sigmoidal (S-shaped) RSOC, where moderate regional recruitment limitation is accompanied by moderate local niche-based filtering. In this intermediate scenario, many species are expected to be widespread. With little disturbance, competitive species are expected to be dominant and widespread, while other species exhibit low occupancy. By contrast, with high disturbance, ruderal species adapted to quick regrowth and dispersal are expected to be widespread, with few late seral specialists. We previously demonstrated that communities in the study region were richest in species at intermediate disturbance [23], so in the current study we adopt Jenkins’ [10] predictions for sigmoidal RSOCs among intermediately disturbed communities, and exponential RSOCs among either low or high disturbed communities. If RSOC shapes are predictive of disturbance, they may be useful indicators of human impacts for management and conservation purposes.

### Specialism and homogenization

The shapes of RSOC’s described above (with some species widespread and others rarely occurring) imply that species vary in the range of conditions under which they may occur. That is, they imply that some species are specialists, and others generalists. Many studies have suggested that human disturbance and invasive species are more likely to negatively affect specialist than generalist species [6,7,9,15,24], including plants [25-27]. Hanski [28] warned that the boreal faces an “imminent wave of extinctions of specialist forest species”. Indeed, specialist species have declined globally at a greater pace than other species [7]. ‘Weedy’, ‘invasive’, ‘generalist’ species are expanding in some areas such that even with declines in specialists, richness may be maintained, if not greater, than in the past [6]. Further, success of introduction and establishment by exotic species is strongly related to generalism [29].

These observations are consistent with theory predicting that generalists should be favored over specialists if human land use acts as disturbance to disrupt spatial homogeneity and/or temporal stability. Niche evolution theory predicts that with less heterogeneity across space, and more stable conditions over time, specialists should be favored [12-14,30,31]. In contrast, with greater spatial and temporal variation, generalists are thought to benefit. Marvier et al. [12] showed this expectation holds despite lower competitive abilities of generalists in any given environment than specialists under the same conditions. These theoretical expectations are explained in part because specialists are thought to be more temporally variable in abundance due to the changing environment [32] and variability in abundance can contribute to extinction [33].

Disturbance can thus be expected to act as a filter for specialist rather than generalist species. However, this hypothesis contrasts expectations from the intermediate disturbance hypothesis, for which generalists are expected to be most prevalent at intermediate, rather than at high disturbance. We predicted that either a) with increasing disturbance, specialism of species would decline assuming that disturbance filters species in favour of those more successful, for example, in higher light conditions and drier soils; or b) that the heterogeneity of disturbance itself influences composition, such specialism should be higher at both low and high disturbance, but generalism higher at intermediate disturbance.

Specialization is usually estimated by either i) laboratory based experimentation on species responses under ranging environmental conditions, or ii) field based observation of niche characteristics and generation of habitat suitability models [7]. Delineating species as specialist, generalist, or in between is in practice challenging with field data because it involves defining the niche of each species in an unknown number of dimensions [34]. Although these dimensions can sometimes be reliably estimated, we instead employ a recently developed index of specialization that bypasses the need to determine niche width directly, and instead uses the diversity of co-occurring species as an indicator of habitat specialization [34]. Specialists are expected to co-occur with similar sets of species wherever they occur, whereas generalists are expected to co-occur with a diverse array of other species. Fridley et al.’s [34] specialization index was reviewed in [7,35] and is related to that of [36]. The index is further explained under Methods. At the community level, we used [34]’s related index as an indicator of functional biotic homogenization, the decline in functional diversity that results from the replacement of specialists by generalists. We answer Clavel et al. [7]’s call that these be used to assess human disturbance on ecological communities.

## Methods

Vascular plant richness was surveyed in the boreal ecoregion of Alberta, Canada. We used data and the standardized protocols of Alberta Biodiversity Monitoring Institute (ABMI) [37], and collected supplementary data at additional sites. In total, vascular plant species occupancy was surveyed within 1 ha for 90 min at a total of 372 sites. We considered all vascular plant species observed at a sampling site to represent an ecological community. Surveys were conducted between Jun 26 and Aug 18 of 2003 to 2011. Human disturbance extent was defined as proportion of land area converted by humans, and was assessed by manual interpretation of 1:30 000 aerial photos and SPOT satellite imagery of a circular area of diameter 1 km at each site, which covers 78.5 ha [38]. Note that the actual ABMI sites, not offset sites, were used for estimating disturbance extent. Only the spatial extent of human disturbance was measured, not its intensity, frequency, or time since disturbance, all of which varied greatly within and among disturbance types. However, the intensity and frequency of disturbance are often related to the extent of disturbance [39,40] including in the boreal forest [41] and expected responses are often similar [42].

### Relative occupancy distributions

To reveal and compare the distribution of species relative occupancy (or ‘dominance’), we created ranked species occupancy curves (RSOCs) following [10]. An RSOC simply ranks species by the number of sites in which it occupies, and plots that occupancy by its rank. RSOCs are akin to occupancy frequency distributions, but do not require binning species by frequency, and relate to ranked species abundance distributions [43,44] but employ presence-absence data rather than abundance data. RSOCs describe the distribution of occupancy across species, and so reveal how widespread species are relative to others in the region, and also how many widespread versus rarely occurring species there are. The shapes of RSOCs have been used to infer community assembly. RSOCs can be fitted to conventional model families (exponential, normal, sigmoidal, linear) and compared [10]. These models can be used to test hypotheses proposed for occupancy frequency distributions, because RSOCs and OFDs represent the same data [10]. However, Jenkins [10] cautions against OFD-based hypotheses because inferring species-specific biological mechanisms like dispersal abilities, niche factors, or metapopulation processes for multiple communities (as in an RSOC or OFD) assumes these mechanisms are similar across species. Instead, he offers community based hypotheses such as succession and intermediate disturbance hypothesis as inferences to be derived from RSOCs. In general, an S-shaped (e.g. sigmoidal) curve suggests a group of communities with some very widespread species and some narrowly distributed species, but few moderately widespread species. By contrast, a more even distribution of species occupancy is suggested by a flatter RSOC with a long, low sloped middle section, and random species occupancy is suggested by a linearly decreasing RSOC.

To describe the relative species occupancy of species across boreal Alberta, we plotted the occupancy versus rank across all sites, and then fit candidate non-linear models to the distribution to determine the model of best fit. To compare the relative species occupancy of communities relative to human disturbance, we first classified each site by five 20% ranges in human disturbance extent, determined the occupancy of each species within the subset of sites in each disturbance class, then plotted an RSOC for each class. We compared candidate models to determine the best fit relationship for each RSOC.

Following [10], we fit exponential decay, asymmetric sigmoidal (cumulative Weibull), and symmetric sigmoidal (logistic) models to the RSOCs in R using the functions ‘nls’ and ‘nls2’ (nonlinear models as defined by [45]). By visual inspection, other common model forms appeared unlikely to improve model fit (normal, linear, etc.). We assessed the significance of difference among models by overlap in confidence intervals. We used analyses of AIC weights to select the best fit model [46,47].

### Species specialization and community homogeneity

To estimate plant species specialization, we used [34]’s specialization index ‘theta’, which is based on co-occurrence among species, rather than species traits or particular environmental conditions. In essence, theta is the mean beta diversity of N sites in which a given species occurs. In using this index, we assume that generalists tend to be found with many diverse species such that species turnover across sites in which they occur is high. Specialists by contrast will typically be found with many of the same species across sites (low turnover). A strength of this method is that niches and their potentially numerous and often unknown axes (*sensu* [11]) need not be defined; co-occurrence acts as an ‘assay’ for diversity of environmental conditions and niche breadth. Since most methods of estimating species specialization rely on predetermining environmental niche axes, and because these axes are often only available for relatively few and more easily measured variables, Fridley’s co-occurrence based specialization can be more practical for large regional datasets [48] like the one used here. The metric accounts for the frequency of species across sites and among habitats to limit influence of sampling design [34,49].

Beta diversity has many formulations [50], and the choice of metric can be critical to assessment of specialization [48,51,52]. Here we use a Beta measure based on variation among all possible pairs of sites, rather than site to site turnover along a directional gradient [50]. We use a multivariate measure of Beta, that compares pairwise similarities in species composition rather than classical measures such as Whittaker’s [53] measure calculated from local and regional diversity directly. We followed [49] and calculated Beta as the mean pairwise Jaccard dissimilarity in species composition of those sites occupied by the focal species, excluding the focal species [54]. Jaccard was calculated with the ‘simba’ package (0.3-5) in R by [55] as: *a*/(2*a* + *b* + *c*) where *a* = number of shared species, *b* = the number of species only found in one of the compared sites, *c* = the number of species only found in the other compared site. This pairwise method was preferred over multiple plot dissimilarity methods [56] because it is insensitive to compositional nestedness, a condition where changes in species loss (subsetting) across a gradient are not considered a change in beta diversity [49].

In calculating the specialization index, the minimum frequency of occurrence for a species to be included must be set. This somewhat arbitrary parameter value must be small enough so as not to exclude too many uncommon species, but large enough that variance from low sample size is not unreasonably high. We selected species with a minimum occurrence of 10 sites, which accounted for 48.1% of all species. Because this minimum occurrence is low, we tested the sensitivity of the parameter to number of sites by repeating the analyses with minimum occurrence of five and two, following [49]. We observed that the density distribution of specialists vs. generalists was not sensitive to low minimum occurrence.

To assess how specialists and generalists relate to disturbance, we plotted richness-disturbance relationships of the 50 species with the highest or lowest Jaccard dissimilarities. We fit linear and quadratic relationships to these plots and selected the models with the lowest AIC values [46].

To assess how species specialism relates to mean disturbance extent across sites, we compared each species’ specialism index value to the mean human disturbance extent across all sites in which the species occurs. If most species were specialists of say, low disturbance levels, Jaccard dissimilarity would be low (i.e. below 0.5) for most species and they would have low mean disturbance extents. If species were all specialized, but for different levels of disturbance (some specialized on old forests, some specialized on intermediately disturbed areas, and others on extensive disturbance), it would be expected that all species would have low Jaccard dissimilarities, but exhibit a flat linear distribution across the gradient in mean disturbance. If species are specialists for a particular disturbance level, it would be expected that Jaccard dissimilarity would increase or decrease with mean disturbance.

The specialism index should not be sensitive to occupancy because such a result would imply species occurring in fewer sites would be more likely to be specialist. In other words, the specialism index is based not on the number of sites in which it is present, but on the species with which it occurs. We plotted the relationship between species specialism and site occupancy to test this fundamental assumption of the specialism index; a significant linear relationship would suggest the assumption was violated. Species were categorized according to their mean disturbance of occupied sites.

At the community level, we assessed the relationship of biotic homogenization to anthropogenic disturbance. We defined biotic homogenization simply as the mean specialization of the group of species occupying a site. This is a modification of Devictor and Robert’s [3] Community Specialization Index, but the index we use here is based on occupancy rather than abundance. For each site, we plotted the mean Jaccard dissimilarity across occupant species by the human disturbance extent of the site. A linear relationship would be expected if sites tended to host relatively more specialist or generalist species according to the extent of site disturbance. A curvilinear relationship would suggest a tendency for sites with intermediate disturbance levels to host either relatively more specialist (if concave down) or generalist species (if concave up).

## Results

**Figure 1:**
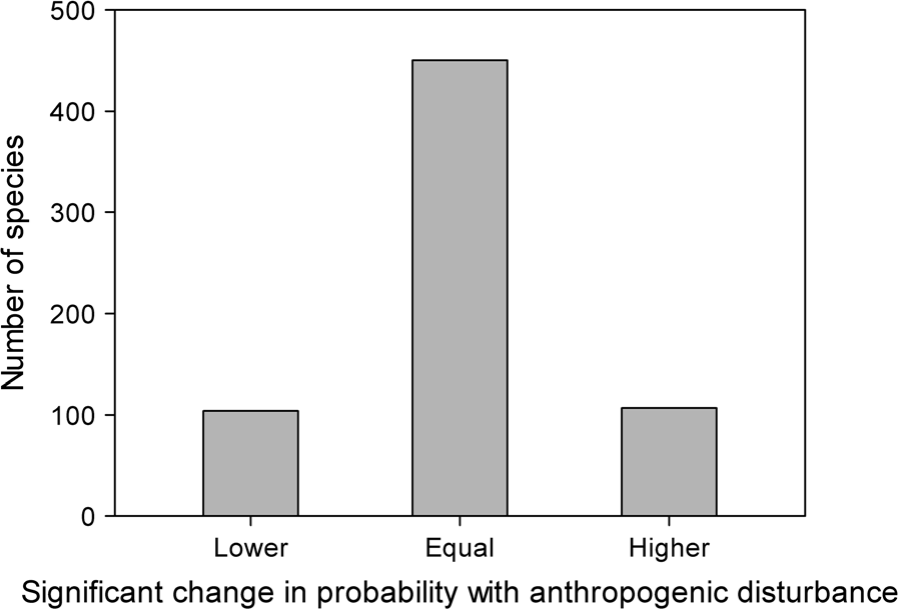
**Frequency histogram of counts of species with significantly lower,** **equal,** **or higher likelihood of occupancy with anthropogenic disturbance extent.**

In 371 sites, 662 species were observed. Of these, 104 species (15.7%) were significantly more likely to be observed in sites of more extensive human disturbance, 107 species (16.2%) were more likely to be observed in sites with less human disturbance, and the presence of each of the remaining 450 species (68.0%) was not significantly related to human disturbance extent (Figure 1).

### Ranked occupancy

**Figure 2:**
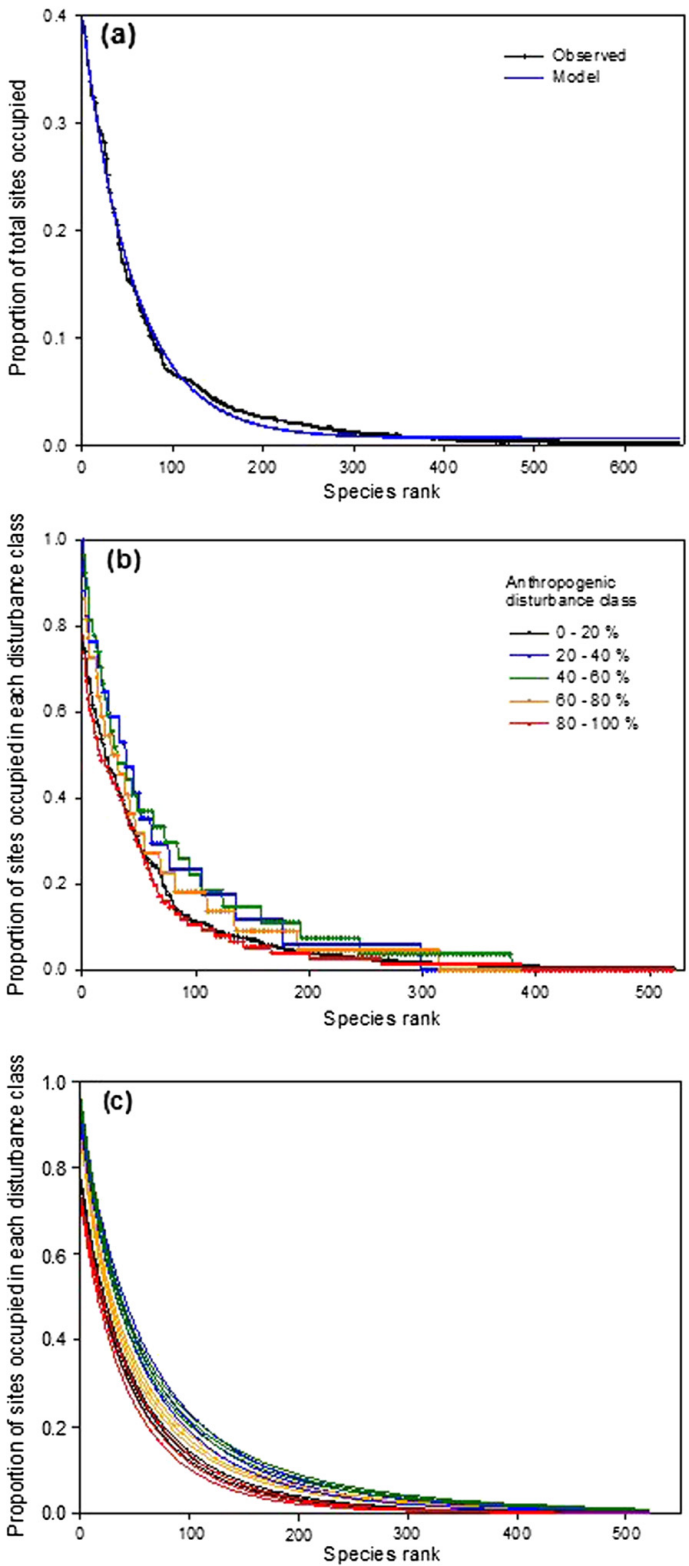
**Ranked species occupancy curves. (a)** Rank occupancy of all species in all sites. Note the shorter y axis than in **(b)** and **(c)**, which indicates many species were typically found in one or few disturbance classes. **(b)** Ranked occupancy of species in each of five subsets of sites classified by percent anthropogenic disturbance. Dots indicate occupancy of each species within each individual land use class. Species are ranked in order of increasing proportion of sites occupied. Occupancy was assessed for each individual disturbance class such that the first ranked species has the greatest occupancy within a given class and may be different across classes. **(c)** Best fit exponential decay models of RSOC distributions in **(a)**. Colours correspond to **(a)**. Thick lines indicate model fit, thin lines indicate 95% confidence intervals, where lack of overlap in intervals indicates statistically significant difference in model.

When all species were ranked by their occupancy and plotted as a ranked species occupancy curve, the curve best fit an exponential decay model. This curve shape shows that a few species are relatively widespread, whereas most species are observed in only very few sites (Figure 2a). The 5% most widespread species occurred in over 30% of sites, the 10% most widespread species occur in at least 12% of sites, whereas the remaining 90% of species occur in less than 12% of sites, and the 50% least widespread species occurred in less than 1% of sites.

When sites were separated by disturbance class, RSOC distributions in each class fit exponential decay models better than other candidate model shapes (in each case, AIC model weight = 1.00, delta = 0 (Figure 2b, c). However, within individual disturbance classes, some species were present in nearly every site, indicating that occupancy is strongly influenced by disturbance class. Comparing the RSOC models for each disturbance class reveals several findings: first, that the shape of this fundamental descriptor of community structure is very similar (exponential) regardless of the extent of disturbance in sites. Second, the most widespread species were very uneven in their occupancy. In each disturbance class the proportion of sites occupied declined rapidly from the most widespread species to the next 50 to 100 most widespread species. For instance, the most prevalent species in sites with 40% to 60% area disturbed was found in 96.3% of those sites, but the 25^th^ most prevalent species was found only in 54.6% of sites and the 50^th^ most prevalent species was only found in 37.0% of sites in that disturbance class. Thus, there were few widespread species, regardless of disturbance class. Third, there were many localized species in each disturbance class. That is, most species were found only in a small proportion of sites, even of a given disturbance class.

Despite the consistently exponential shapes of occupancy curves, RSOCs were statistically distinguishable across disturbance classes (Figure 2c), indicating differences in evenness of occupancy across species when comparing disturbance classes. In general, species in sites of intermediate disturbance classes were more even in their occupancy and more widespread than species in high and low disturbance extent communities. More species were observed at higher proportions of sites in intermediate disturbance classes than at either high or low disturbance classes. The lowest (0 – 20% disturbance) and highest (80 – 100%) disturbance classes overlapped. The occupancy strructure of species in the low-mid to middle disturbance classes also overlapped, but species occupancy was generally higher and more even across species. (i.e. less right skewed). The mid-high disturbance class was less even than those but more even than the low and high disturbance classes.

RSOC models among disturbance classes tended to converge toward high (>300) and very low (<25) ranked species; differences in the model distributions were most apparent among ranks of 50 to 300 species (Figure 2c). This observation indicates that the most substantial differences in community structure across disturbance classes were in the occupancy proportions of moderately widespread species.

Ranking of species by occupancy also permitted the identities of high-occupancy species to be compared in each disturbance class (Table 1). Species like *Taraxum officinale* and *Achillea millefolium*, typically associated with disturbed or high light conditions, were found (as expected) at high occupancy in high disturbance classes, whereas species like *Cornus canadensis* and *Linnaea borealis*, typical forest understory species, were found at high occupancy only in low disturbance classes. On the other hand, some species like *Populus tremuloides* ranked at high occupancy across the range of disturbance classes.

**Table 1:** **List of species ranked by occupancy for each disturbance class** (**given as percentages**)

**Rank**	**0** **-** **20%**	**20** **-** **40%**	**40** **-** **60%**	**60** **-** **80%**	**80** **-** **100%**
1	Cornus canadensis	Populus tremuloides	Achillea millefolium	Equisetum arvense	Taraxacum officinale
2	Rhododendron groenlandicum	Galium boreale	Fragaria virginiana	Vicia americana	Achillea millefolium
3	Chamerion angustifolium	Cornus canadensis	Populus tremuloides	Rubus idaeus	Equisetum arvense
4	Vaccinium vitis-idaea	Equisetum arvense	Taraxacum officinale	Taraxacum officinale	Vicia americana
5	Calamagrostis canadensis	Rosa acicularis	Vicia americana	Populus tremuloides	Fragaria virginiana
6	Rosa acicularis	Calamagrostis canadensis	Equisetum arvense	Achillea millefolium	Galium boreale
7	Populus tremuloides	Chamerion angustifolium	Galium boreale	Fragaria virginiana	Populus balsamifera
8	Linnaea borealis	Fragaria virginiana	Picea glauca	Mertensia paniculata	Rosa acicularis
9	Petasites frigidus	Lathyrus ochroleucus	Populus balsamifera	Picea glauca	Rubus idaeus
10	Rubus pubescens	Mertensia paniculata	Chamerion angustifolium	Populus balsamifera	Populus tremuloides

Species listed represent the ten most “widespread” species in the Ranked Species Occupancy Curves of Figure 2(b).

### Specialization and homogeneity

**Figure 3:**
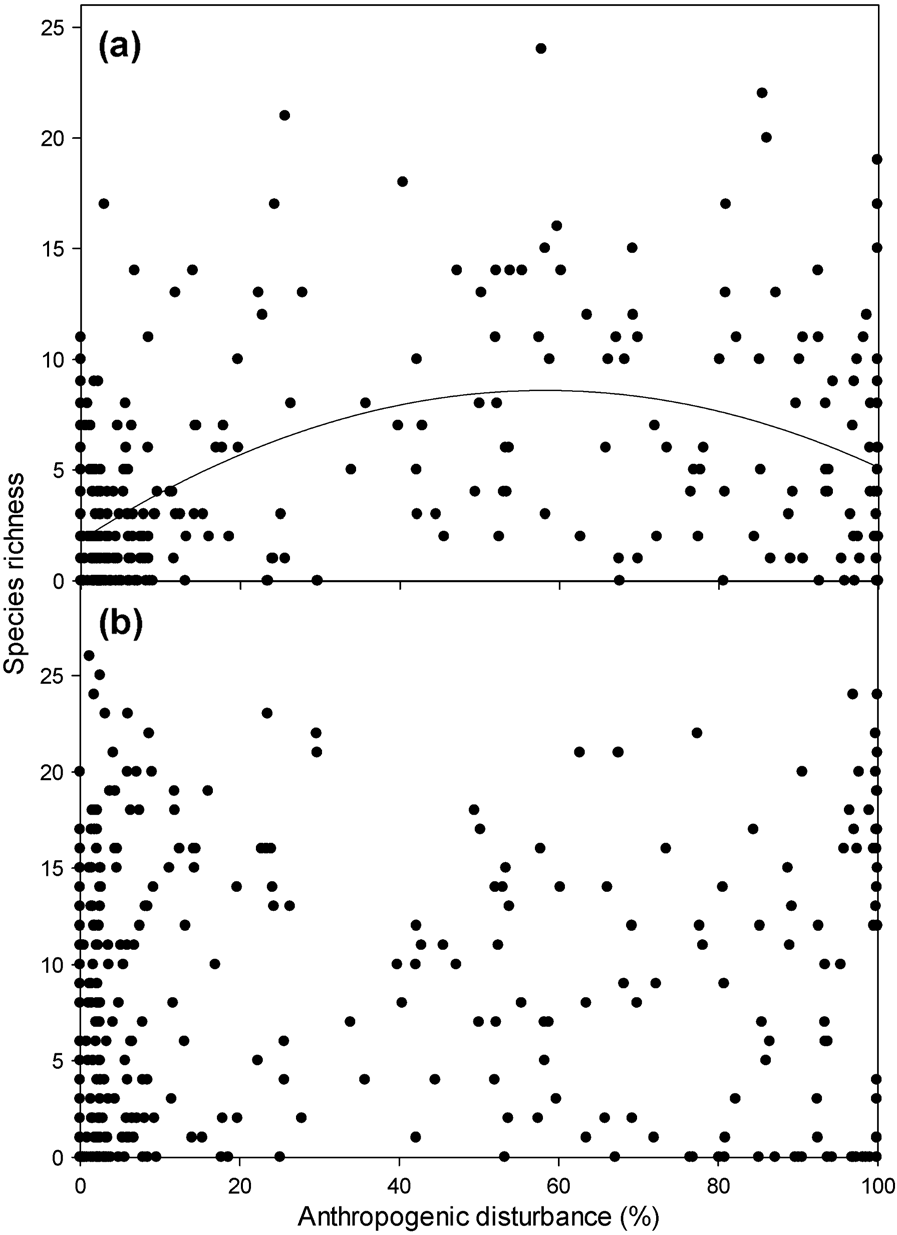
**Species richness of generalists and specialists relative to percent anthropogenic disturbance extent. (a)** The 50 most ‘generalist’ species observed (y = 0.00199x2 + 0.232x + 1.855, R2 = 0.242, p < 0.001), and **(b)** the 50 most ‘specialist’ species (y = 0.000922x2 + 0.0970x + 7.177, R2 = 0.010, p = 0.050). Best fit polynomial regression line shown in **(a)**, no ecologically significant trend for **(b)**.

Richness of the 50 most generalist species was highest at intermediate human land use (Figure 3a, y = 0.00199x^2^ + 0.232x + 1.855, R^2^ = 0.242, p < 0.001). The same cannot be concluded for the most ‘specialist’ species. The richness of the 50 most specialist species related to disturbance with borderline statistical significance, but the relationship was very weak and not ecologically significant (Figure 3b, y = 0.000922x^2^ + 0.0970x + 7.177, R^2^ = 0.010, p = 0.050).

**Figure 4:**
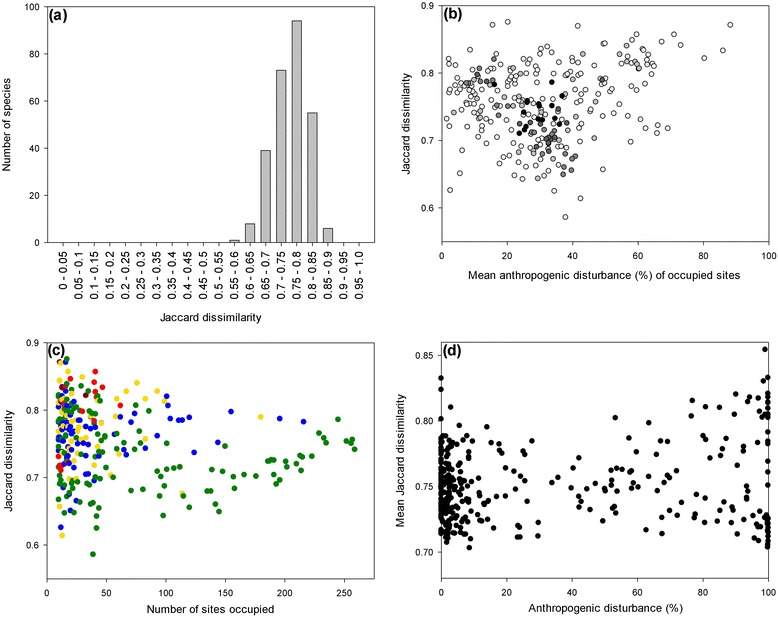
**Vascular plant species specialism and anthropogenic disturbance extent** **(%).** Jaccard dissimilarity is an index of ‘specialism’ (lower values) to ‘generalism’ (higher values) ranging from 0 to 1. It is calculated based on species co-occurrence; in effect it measures turnover in co-occupants experienced by the focal species. **(a)** Frequency histogram of species specialism. Species with high Jaccard dissimilarity co-occur with a wide variety of other species. No species were observed with low dissimilarity, indicating little fidelity to co-occurring species. **(b)** Species generalism as a function of the mean anthropogenic disturbance extent across sites occupied by the focal species. Dots represent individual species and darker shading indicates species occurring in more sites. **(c)** Species generalism relative to site occupancy. Dots represent species, and warmer colours indicate species with higher mean anthropogenic disturbance in occupied sites. **(d)** Community homogeneity relative to percent anthropogenic disturbance extent. Each dot represents a site, and the mean Jaccard dissimilarity is the average Jaccard index across species occurring at that site. Note that the scales differ for **(b)** - **(d)**.

The Jaccard dissimilarity index of niche breadth varies from 0 to 1. Low numbers indicate a species which co-occurs with a very small set of other species, a pattern suggesting habitat specialization; we call these species ‘specialist’ following Fridley et al. [34] and as discussed by Devictor et al., [35]. The converse, high Jaccard dissimilarity indicates a species which co-occurs with a wide variety of other species, here called ‘generalist’s. ‘Niche breadth’ varied between 0.586 and 0.875, indicating that all species observed exhibited wide co-occurrence based ‘niche breadth’ indices: all species appear to be moderate ‘generalists’ (Figure 4a). Species ‘niche breadth’ had a mean of 0.755, and a median of 0.757.

Species ‘generalism’ was not strongly related to mean anthropogenic disturbance extent of sites occupied by a species. Jaccard dissimilarity of co-occurring species was statistically significantly related to mean disturbance (Figure 4b, y = 0.780 - 0.00267x + 0.0000449x^2^, R^2^ = 0.106, p < 0.001), but this relationship was somewhat weak and sensitive to outliers (at high disturbance) so we consider it ecologically non-significant. Thus, generalists were not found disproportionately at sites of higher disturbance. ‘Generalism’ was also not substantially related to the number of sites occupied by a species, despite statistical significance (Figure 4c, y = 0.774 + 0.000537x, R^2^ = 0.0382, p < 0.001). This confirms that Jaccard dissimilarity is not very sensitive to occupancy; generalists don’t occupy substantially more sites.

Community biotic homogeneity was likewise not strongly related to human disturbance extent (Figure 4d, y = 0.746 + 0.000146x, R^2^ = 0.0373, p < 0.001), noting again the statistical significance. Each of these analyses included only species with at least 10 occurrences to ensure ecologically meaningful assessment of co-occurrence. However, each of these analyses were repeated with all species with at least 2 occurrences, and the results were similarly weak; no conclusions changed.

## Discussion

Declining biodiversity and associated biotic homogenization are among the chief global conservation concerns [6,7,17,57]. The decline of specialists with disturbance, and their replacement by generalists is expected to lead to more homogeneous communities [7,57,58], both in terms of composition and function. Clavel et al. [7] recommended using the level of specialization and homogeneity in a community to gauge the impacts of disturbance on biodiversity. For example, Devictor et al. [9] showed that the mean habitat specialization of birds declined with human disturbance, concluding that communities were more functionally homogeneous with disturbance.

With increasing anthropogenic disturbance, most species (68.0%) were no more or less likely to occur in a given site. Nearly an equal proportion of species were more likely as were less likely to occur with increasing human disturbance. This balance in ‘increasers’ and ‘decreasers’ with disturbance is consistent with our earlier findings [23] supporting the intermediate disturbance hypothesis in that low and high disturbance sites were similar in species richness. It is also consistent with Devictor et al. [15], who on a similar regional scale observed that roughly half of bird species throughout France increased with disturbance while half decreased. On the basis of species richness alone, it can appear that human disturbance is relatively benign.

We expected disturbance to alter assembly, and therefore occupancy structure (RSOCs) of communities. Specifically, we expected exponential RSOC among communities with either low or high disturbance, due to the effects of competitive exclusion and recruitment limitation, respectively, and a sigmoidal RSOC among intermediately disturbed communities, where recruitment limitation and competition are both expected to be moderate [10]. Species occupancy structure was statistically distinguishable among disturbance classes, with species slightly more even in their occupancy of intermediately disturbed sites than among sites of low or high disturbance (Figure 2c). That is, there were more species that were moderately prevalent among intermediately disturbed communities. However, the general structure of communities in these disturbance classes was equivalent in shape; regardless of disturbance extent, species occupancy decayed exponentially from most to least prevalent species. That is, only a few species occurred in a high proportion of communities, regardless of disturbance. This general observation is supported by [59] who reported similar ranked species abundance distributions for boreal plants in sivicultural landscapes. However, these findings somewhat contrast the findings of [10] who suggested that RSOCs should exhibit a sigmoidal shape at intermediate disturbance. This suggests that RSOC shapes may not be as consistently predictable across metacommunities as Jenkins [10] proposed [60], despite the support for the intermediate disturbance hypothesis [23] that forms the basis for the predictions. Ranked species occupancy curves may be expected to be more exponential at large spatial scales and more sigmoidal at smaller spatial scales [10], which could explain the observed consistency in exponential RSOCs observed in this large scale study. However, the scale dependence of RSOC shape has little empirical support [10].

The weak impact of disturbance on occupancy structure to disturbance was mirrored by weak relationships of species specialization to disturbance. Contrasting [9] but consistent with [61] and [62], specialization was not strongly related to human disturbance. Specialist species were expected to be among the most sensitive groups to human disturbance [35] following one of two related theoretical approaches. Either specialists should decline with disturbance if disturbance creates spatially and temporally heterogeneous environmental conditions more favorable to generalists [9,13,14,30], or specialists should exhibit greater occupancy at very low and very high disturbance if these areas are unique but internally more homogeneous [18,63]. We found, on the contrary, that the richness of the most specialized species was only marginally statistically related to disturbance extent. The most generalist species on the other hand fit a quadratic model with higher richness at intermediate to high disturbance.

All the species we observed were identified as generalists according to the co-occurrence based specialization index (Figure 4a). This means all species co-occurred with a wide variety of species—none showed fidelity to a small subset of species, despite variation in generalism. The apparent ubiquity of boreal plant species generalism may be due to: i) extreme seasonal variation in climatic conditions, ii) relatively short time duration since glaciation [64], or iii) failure of the co-occurrence based index of specialization used in this study to identify true specialists. These findings suggest that in this system, specialization is unlikely a key mechanism of niche differentiation facilitating species coexistence [65]. This failure to identify specialists might have been an artifact of relatively large 1 ha sample sites, however, the boreal region is large-grained [66] relative to highly specialized systems like tropical forests, suggesting the sampling scheme was appropriate. It is surprising that theoretical frameworks developed in more heterogeneous, highly specialized systems did not apply to the boreal, even recognizing in advance that the boreal is an ecosystem with relatively low specialization, high species turnover, and species with high propensities for dispersal.

A species’ degree of specialization was not strongly related to the mean disturbance of the sites it occupied (Figure 4b-c). Functional biotic homogenization of communities was similarly unrelated to anthropogenic disturbance (Figure 4d). The richness of even the least generalized species was unrelated to disturbance (Figure 3). These results appear to contrast previous studies that have suggested specialist species are increasingly shown to be experiencing higher rates of decline and extinction, part of an emerging pattern of biotic homogenization of communities becoming more similar to each other [7,15,17,67-71]. Christian et al. [6] observed that over a century-long time series of insular birds, species richness increased while specialist richness decreased. Even paleontological data has shown that specialists were disproportionately prone to extinction in previous mass extinctions [72,73]. Vázquez & Simberloff [62] however refuted the idea that specialization increases with disturbance and two studies found the opposite pattern: bird communities in disturbed habitats were more specialized [74,75].

## Conclusions

Overall, the results of this study imply relatively little sensitivity to even very extensive (i.e. 100% coverage of) human land use in the boreal forest vascular plant communities. This apparent insensitivity of community structure to disturbance might be explained by the high generalism of most species, particularly relative to highly specialized systems like tropical forests e.g. [76,77] or along strong environmental gradients such as alpine slopes [48]. Are communities truly impacted so little by disturbance, or are these findings idiosyncrasies of this study? Several non-mutually exclusive possibilities exist. First, communities may be resilient or resistant to human disturbance in this boreal region and face little risk. Second, communities may be sensitive to human disturbance, but in ways not captured by the metrics used in the current study’s analyses. Third, communities may be sensitive to the metrics explored, but depend on an alternative sampling strategy.

## Abbreviations

ABMI: Alberta Biodiversity Monitoring Institute
OFD: Occupancy frequency distribution
RSOC: Ranked species occupancy curve
SPOT: Satellite Pour l’Observation de la Terre (Satellite for observation of Earth)
